# Supplementary material for: MARCH8 inhibits viral infection by two different mechanisms
Source: eLife. 2020 Aug 11;9:e57763. doi: 10.7554/eLife.57763 (PMC7419139; doi:10.7554/eLife.57763)
Supplement: Source data 2. — Original uncropped images of IP-western blot (ubiquitination assays) in Figure 1G. The PVDF membranes were incubated with an anti-T7-epitope tag antibody, or with an anti-ubiquitin antibody. Images shown in Figure 1G were cropped from the boxed areas, and the brightness/contrast was adjusted equally across the entire image using Photoshop CS6 Figure 2B source data. Original uncropped images of western blot in Figure 2B. The PVDF membrane was incubated with an anti-HA antibody, then stripped and reprobed with an anti-β-actin antibody for a loading control. Images shown in Figure 2B were cropped from the boxed areas, and the brightness/contrast was adjusted equally across the entire image using Photoshop CS6. [file elife-57763-data2.pdf]

Source data

MARCH8 inhibits viral infection by two different mechanisms

Yanzhao Zhang, Takuya Tada, Seiya Ozono, Satoshi Kishigami,
Hideaki Fujita, and Kenzo Tokunaga

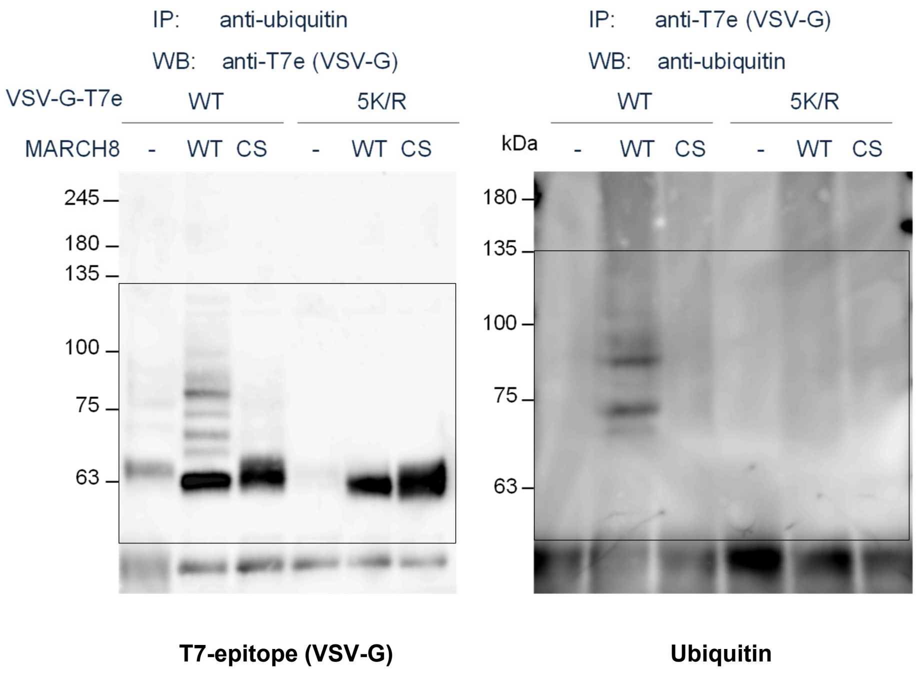

Figure 1G-source data.

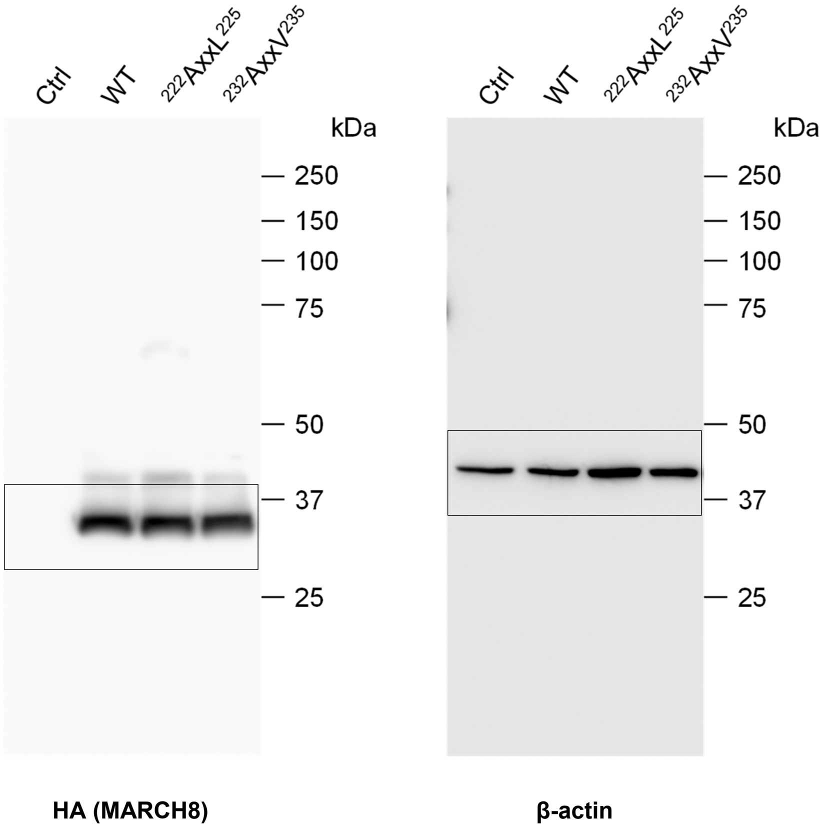

Figure 2B-source data.
